# Hope and great opportunity for young neuroscientist

**DOI:** 10.12669/pjms.294.3944

**Published:** 2013

**Authors:** Shahid Bashir

**Affiliations:** 1 Shahid Bashir, Division of Cognitive Neurology, Berenson-Allen Center for Noninvasive Brain Stimulation, Department of Neurology, Beth Israel Deaconess Medical Center, Harvard Medical School, Boston, MA, USA.; 2 KSU-Autism Research and Treatment Center, Department of Physiology, Faculty of Medicine, King Saud University, P O Box 2925, Riyadh 11461, Saudi Arabia.

Understanding the human brain is one of the greatest challenges facing 21st century science. For a long time though, it has been a closed black box to us. Different strategies have been employed in trying to unravel the mysteries inside the black box. On this basis, the goal is to discover more and more complex structures until a complete understanding of the brain is achieved.

According to a recent study^1^ majority of Asian citizens are affected by some kind of brain disease, the most frequent being headache, anxiety disorders, sleep disorders and mood disorders.

In the recent years a revolution in neuroscience has taken place. New tools for imaging the brain such as functional magnetic resonance imagining (fMRI), Electroencephalography (EEG), Magnetoencephalography (MEG), Positron emission tomography (PET), and Single-photon emission computed tomography (SPECT) have provided us with a way to investigate the human brain while the subject performs a variety of tasks^2^. Today the diagnostic use of EEG in a clinical setting is for epilepsy as well as many other areas such as sleep-disorders, strokes, infectious diseases, brain tumors, mental retardation, severe head injury, drug overdose, brain death, etc.^3^).

Today, diseases of the brain are usually diagnosed in terms of symptoms and syndromes, an approach that makes it very difficult to produce definitive diagnoses, or to select the patients who will benefit from a particular therapy. Similarly, most therapy is designed to manage symptoms, with only very few treatments attempting to modify the course of the underlying disease. Studies have shown that conditions classified as a single disease can have completely different genetic causes and that diseases with different causes can produce very similar symptoms. 

These are good times in neuroscience. Almost simultaneously, the European Union and United States have raised a similar challenge for the next decade: still lot of progress is needed to get to understand the human brain. The Human Brain Project (HBP) of the European Union, and Brain Activity Map (BAM), United States are two ambitious projects underway in this regard.

The European project, The Human Brain Project aims to create a new platform or instrument, a kind of artificial brain simulator that allows a substantial jump methodology for advancing neuroscience, medicine and information technology of the future. It would be a completely new way of doing things and clinical information generated in this project could be used to design new forms of artificial intelligence and more powerful computers.

The American project, Brain Activity Map, precisely focuses on critical basic information on the brain which is still lacking, to determine how the brain works when it is in full swing, and the functional properties that arise from the interaction of brain circuits that give rise to the emergence of mental functions?

Researchers (neuroscientists and neurologists) around the world and especially in Asia have their greatest opportunities to join in and discover the unknowns in the brain because a momentum of unprecedented opportunities in the Asian region has been generated in recent years. In this setting, a vibrant group of universities can also bring improvements in the region that can permeate to the society and set the platform for young neuroscientist. As we know in Asia, there is an urgent need to improve diagnosis, better characterize, promote prevention and/or provide treatment in various number brain pathologies: neurodegenerative diseases, neuropsychiatric disorder, neurologic conditions etc.

## References

[1] Ayuso-Mateos, Jose Luis "Global burden of panic disorder in the year 2000".

[2] Pascual-Leone A, Freitas C, Oberman L, Horvath JC, Halko M, Eldaief M (2011). Characterizing brain cortical plasticity and network dynamics across the age-span in health and disease with TMS-EEG and TMS-fMRI. Brain Topogr.

[3] Nunez, PL, Srinivasan R ([Preview] 2006). Electric Fields of the Brain: The Neurophysics of EEG.

